# Taking the Pulse of POCUS: The State of Point-of-Care Ultrasound at a Pediatric Tertiary Care Hospital

**DOI:** 10.24908/pocus.v6i2.14781

**Published:** 2021-11-23

**Authors:** Peter Gutierrez, Tal Berkowitz, Lekha Shah, Stephanie G Cohen

**Affiliations:** 1 Emory University School of Medicine Atlanta, GA; 2 Children's Healthcare of Atlanta Atlanta, GA

**Keywords:** Pediatrics, Education

## Abstract

We aim to quantify and categorize point-of-care ultrasound (POCUS) usage by pediatric practitioners and trainees at our tertiary care center, and assess the degree of interest from pediatric residents, fellows, and program leaders for integrating POCUS into their training. Data was collected via online survey, evaluating the current use of POCUS in clinical decision making, desire for further formal training, and opinions on the importance of POCUS to future clinical practice. In total, 14 program directors/assistant program directors (PD/APDs) representing 10 of 15 training programs, 30 of 95 fellows representing 9 of 15 fellowships, and 32 of 82 residents responded. From PD/APDs, only 2 of the programs reported active use POCUS for clinical decision making, but 13 of the fellows and 9 residents reported doing so. In regard to desire for a formal POCUS program, 30.8% of PD/APDs, 43.8% of fellows without current curricula, and 87.5% of residents were interested in participating in such a program. When considering specialty, some non-acute care-based PD/APDs and fellows at our institution felt that POCUS was important to future practice. Pediatric subspecialty PD/APDs and their fellows had divergent outlooks on the importance of POCUS in future practice. Finally, an overwhelming majority of residents at our institution expressed a desire to learn, and half believing it will be important to future practice. Based on the degree of interest, medicolegal considerations, and trajectory of patient care, pediatric residency and fellowship programs should strongly consider integrating POCUS education into their curricula.

## Background

Point-of-care ultrasound (POCUS), ultrasound both performed and interpreted by a clinician at the bedside and used to answer immediate questions or to perform procedures, is becoming an integral part of daily practice in many areas of medicine. Most published curricula exist in adult-focused specialties, including emergency medicine (1, 2, 3), internal/family medicine 4, 5, 6, 7 critical care 8, general surgery 9, anesthesiology 10, and nephrology 11. There are few similar publications in pediatric specialties, with pediatric emergency medicine and critical care being notable exceptions 12, 13, 14, 15. While POCUS is most consistently used in the acute care setting, it also has applications in the ambulatory and inpatient settings 16. 

To our knowledge, there are no published reports of the degree of POCUS use in pediatric subspecialties. Currently, there are multiple pediatric subspecialties that have board content specifications that involve ultrasound, but the majority involve only knowing its indications or limitations (adolescent medicine, child abuse pediatrics, neonatology), interpretation of scans performed by technicians or radiologists (gastroenterology, plastic surgery), or have very limited applications (cardiology). Other subspecialties express only an awareness of the modality (endocrinology, neurology/epileptology, neurosurgery). Pediatric emergency medicine (EM) is unique in that its board content specifications explicitly mentions POCUS 17, 18, 19, 20, 21, 22, 23, 24, 25, 26, 27. In spite of this, multiple specialties at our institution report the use of POCUS in clinical decision-making, which prompted our inquiry. The primary purpose of this study is to quantify and categorize the use of POCUS by pediatric subspecialists across our large tertiary care academic pediatric hospital system. Secondary aims of this study were to assess the degree of interest in formal POCUS training and perceived importance to future clinical practice by pediatric trainees and their program leaders.

## Methods

Data from this study was collected via an online survey. One survey type was sent to the program directors and associate program directors (PDs/APDs) for the pediatric residency and pediatric fellowships at our institution. A separate, but similar, survey sent to the general pediatric residents and subspecialty fellows in training at our institution at the time of administration. Surveys listed demographics, training level, questions about current practice and future training interest in POCUS, and attitudes about the importance of POCUS in future clinical practice. Surveys were created in REDCAP and disseminated via email. Results were de-identified and underwent descriptive statistical analysis upon completion of the study period. Current PD/APDs of general pediatrics or a pediatric subspecialty, general pediatric residents, or pediatric subspecialty fellows were included. There were no exclusion criteria. Data was compiled on secure SharePoint and REDCAP sites. Statistical analyses were performed using Microsoft Excel (Microsoft Excel for Mac 2019, Microsoft Corporation, Version 16.34), which included calculating percentages. This study was granted exemption from our institutional review board.

## Results

In total, 14 PDs/APDs responded to the survey, representing 10 of the 15 pediatric fellowship programs available at our institution. Respondents included representatives from endocrinology, cardiology, gastroenterology (GI), nephrology, allergy/immunology, hematology/oncology, rheumatology, neurology, neonatology, and critical care medicine (CCM). Only two of the respondents, a rheumatologist and a cardiologist, indicated that they personally perform point-of-care ultrasound scans for medical decision-making. The rheumatologist reporting using point-of-care (POC) musculoskeletal imaging, and the cardiologist reported using POC noncardiac thoracic imaging. Regarding the importance of POCUS in future practice of their specialty, four respondents indicated it was “important” and the remaining ten were either neutral or reported it was not important. Currently, only one program (CCM) reported having a current POCUS curriculum for their fellows. Of note, the program director for EM did not respond, but that specialty is known to have a formal curriculum for its fellows. Four of 13 respondents without a POCUS program, cardiology, nephrology, endocrinology, and neonatology, reported a desire to create a POCUS course for their fellows (Table 1).

**Table 1:** Responses from PD/APDs to POCUS Survey

**Question**	**Response Choice**	**Response No. (%)**	**Specialty**
Number of respondents		14	
Program Directors		7 of 14 (50%)	
Program represented	Allergy/immunology (A/I)	1	
	Cardiology	2	
	Critical Care Medicine (CCM)	1	
	Endocrinology	2	
	Gastroenterology (GI)	1	
	Hematology/Oncology (H/O)	2	
	Neonatology	1	
	Nephrology	2	
	Neurology	1	
	Rheumatology	1	
Years in practice	Less than 5 years	3 of 14 (21.4%)	
	5 to 10 years	5 (35.7%)	
	10 to 15 hours	2 (14.3%)	
	more than 15 years	4 (28.6%)	
Years in PD/APD role	Less than 5 years	9 of 14 (64.3%)	
	5 to 10 years	3 (21.4%)	
	10 to 15 hours	2 (14.3%)	
	more than 15 years	0 (0%)	
Currently use POCUS in clinical practice		2 of 14 (14.3%)	Cardiology, Rheumatology
Modality used in current practice	Thoracic	1 of 14 (7.1%)	Cardiology
	Musculoskeletal	2 (14.3%)	Rheumatology
	All others	0	
POCUS modalities beneficial for present/future practice	All modalities	0 of 14 (0%)	
Anticipating importance of POCUS in future practice	Very Important	0 of 14 (0%)	
	Important	4 (28.6%)	Cardiology, Endocrinology, Nephrology, Rheumatology
	Neutral	5 (35.7%)	A/I, Cardiology, CCM, H/O, Neonatology
	Not Important	3 (21.4%)	Endocrinology, Nephrology, Neurology
	Not Important at all	2 (14.3%)	GI, H/O
Currently have a POCUS training curriculum		1 of 14 (7.1%)	CCM
Planning on creating a POCUS training curriculum		0 of 14 (0%)	
Interest in having trainees participate in POCUS curriculum		4 of 13 (30.8%)	Cardiology, Nephrology, Endocrinology, Neonatology
*Note: Denominator excludes CCM, which already had a curriculum.			
POCUS quality assurance program in place		0 of 14 (0%)	

Thirty out of 95 fellows representing 9 of 15 total fellowships responded to the survey. Regarding types of scans being performed for clinical decision making, thirteen respondents reported using POCUS clinically to some degree while 17 reported not using it at all. All applications except airway, thyroid, abdomen, and male genitourinary (GU) were reported as currently used. When CCM and EM fellows were removed, only cardiac, aorta, renal/bladder, MSK, soft tissue, and thoracic modalities were reported. Remaining reports were from cardiology fellows, except for one MSK respondent from an undeclared specialty. When asked what applications fellows wanted to learn, respondents from nine different specialties listed at least one application they felt would be beneficial to their practice. With regards to importance of POCUS to the future of their specialty, 16 of 30 responded “important” or “very important”, while 14 responded neutral or not important. Currently, only critical care medicine, emergency medicine, and cardiology respondents reported having a POCUS curriculum. Of note, there were two respondents from cardiology, one of whom reported that their program had a formal POCUS curriculum, while other reported that there was not one. Of the 18 non-EM/CCM fellow respondents that are known to not have a POCUS curriculum, eight would like to start a program, including those from endocrinology, GI, hospitalist medicine, neonatology, two that did not report their training program, and the other respondent from cardiology (Table 2).

**Table 2:** Responses from subspecialty fellows to POCUS survey. (Continues on next page)

**Question**	**Selection**	**Response No. (%)**	**Subspecialty**
Number of respondents		30 of 95 (31.6%)	
Programs represented	Cardiology	2	
	Critical Care Medicine (CCM)	5	
	Emergency Medicine (EM)	7	
	Endocrinology	4	
	Gastroenterology (GI)	2	
	Hematology/Oncology (H/O)	3	
	Hospitalist Medicine (HM)	1	
	Neonatology	2	
	Nephrology	1	
	Unreported	3	
Post-graduate year	4	7 of 30 (23.3%)	
	5	13 (43.3%)	
	6	9 (30.0%)	
	7	1 (3.3%)	
Currently use POCUS in clinical practice		13 of 30 (40.6%)	Cardiology, CCM, EM, Unreported
Modality used in current practice	Aorta	2 of 30 (6.7%)	Cardiology, EM
	Biliary	1 (3.3%)	EM
	eFAST	7 (23.3%)	EM
	Focused Cardiac	5 (16.7%)	Cardiology, CCM, EM
	Musculoskeletal	5 (16.7%)	EM, Unreported
	Obstetric	2 (6.7%)	EM
	Ocular	3 (10.0%)	EM
	Renal/Bladder	5 (16.7%)	Cardiology, CCM, EM
	Soft tissue	9 (30.0%)	Cardiology, CCM, EM
	Thoracic	3 (10.0%)	Cardiology, CCM, EM
	Vascular/DVT	3 (10.0%)	CCM, EM
	Other Modalities	0	
	Do not perform POCUS	17 (56.7%)	
POCUS modalities fellows want to learn	Abdomen	11 of 30 (36.7%)	CCM, EM, GI, HM, Unreported
*Note: Denominator is number of fellows that don't already know the modality*	Airway	8 of 30 (26.7%)	Cardiology, CCM, EM, Neonatology, Unreported
	Aorta	3 of 28 (10.7%)	CCM, EM
	Biliary	2 of 29 (6.9%)	EM
	eFAST	6 of 23 (26.1%)	Cardiology, CCM, EM
	Focused Cardiac	9 of 25 (36.0%)	CCM, EM, Neonatology, Unreported
	Male Genitourinary	3 of 30 (10.0%)	EM, Endocrinology
	Musculoskeletal	4 of 25 (16.0%)	EM, H/O
	Obstetric	3 of 28 (10.7%)	EM, Endocrinology
	Ocular	5 of 27 (18.5%)	EM, Unreported
	Renal/Bladder	5 of 25 (20.0%)	CCM, EM, Nephrology
	Soft tissue	4 of 21 (19.0%)	H/O, HM, Unreported
	Thoracic	6 of 27 (22.2%)	CCM, EM, Neonatology
	Thyroid	4 of 30 (13.3%)	Endocrinology
	Vascular/DVT	8 of 27 (29.6%)	CCM, EM, H/O, Neonatology, Unreported
Anticipating importance of POCUS in future practice	Very Important	8 of 30 (26.7%)	CCM, EM, Unreported
	Important	8 (26.7%)	Cardiology, CCM, EM, Endocrinology, HM, Unreported
	Neutral	7 (23.3%)	Cardiology, CCM, Endocrinology, GI, H/O, Neonatology, Unreported
	Not Important	4 (17.4%)	Endocrinology, GI, Neonatology, Nephrology
	Not Important at all	3 (10.0%)	Endocrinology, GI, H/O
Programs with a POCUS training curriculum	Yes	4 of 30 (13.3%)	Cardiology, CCM, EM, Unreported
Interest in participating in POCUS Curriculum	Yes	7 of 16 (43.8%)	Endocrinology, GI, Neonatology, Unreported
*Note: Denominator is number of fellows from programs that don’t already have a program*			

The survey administered to residents included 32 completed surveys from a possible 76 categorical general pediatric (GP) residents and 6 General Pediatric/Neurology (GP/N) residents, of which 17 came from GP, 3 from GP/N, and 12 unreported. Despite no formal training program, nine residents did report currently using a variety of POCUS modalities, most commonly soft tissue. When asked about which POCUS modalities they would like to learn, all modalities were mentioned by at least one resident, but the most common responses were extended focused assessment with sonography for trauma (eFAST), cardiac, MSK, soft tissue, and abdomen. Half of the residents reported POCUS is either “important” or “very important” to their future learning, while the other half felt it is either “not important” or are “neutral.” (Table 3). 

**Table 3:** Responses from residents to POCUS survey.

**Question**	**Selection**	**Response No. (%)**
Number of respondents		32 of 82 (39.0%)
Programs represented	General Pediatrics (GP)	17 of 32 (53.1%)
	General Pediatrics/Neurology (GP/N)	3 (9.4%)
	Unreported	12 (37.5%)
Post-graduate year	1	6 of 32 (18.8%)
	2	14 (43.8%)
	3	11 (34.4%)
	4 or higher	1 (3.1%)
Currently use POCUS in clinical practice	GP	5 of 32 (15.6%)
	GP/N	0
	Unreported	4 (12.5%)
Modality used in current practice	Abdominal	2 of 32 (6.3%)
	Biliary	1 (3.1%)
	eFAST	2 (6.3%)
	Musculoskeletal	1 (3.1%)
	Soft tissue	6 (18.8%)
	Other Modalities	0
POCUS modalities beneficial for present/future practice	Abdomen	19 of 32 (59.4%)
*Note: Denominator unchanged since residents have no formal POCUS training*	Airway	11 (34.4%)
	Aorta	4 (12.5%)
	Biliary	6 (18.8%)
	eFAST	16 (50.0%)
	Focused Cardiac	13 (40.6)
	Male Genitourinary	7 (21.9%)
	Musculoskeletal	14 (43.8%)
	Obstetric	4 (12.5%)
	Ocular	5 (15.6%)
	Renal/Bladder	12 (37.5%)
	Soft tissue	16 (50.0%)
	Thoracic	7 (21.9%)
	Thyroid	3 (9.4%)
	Vascular/DVT	11 (34.4%)
Anticipating importance of POCUS in future practice	Very Important	5 of 32 (15.6%)
	Important	11 (34.4%)
	Neutral	8 (25.0%)
	Not Important	6 (18.8%)
	Not Important at all	2 (6.3%)
Interest in participating in POCUS curriculum	Yes	28 of 32 (87.5%)

## Discussion

Within our institution there is currently formal POCUS training limited to two specialties, EM and CCM, with some formal teaching in cardiology in addition to their echocardiography training, but not an official POCUS curriculum. 

Outside of EM, CCM, cardiology, and rheumatology, faculty did not have experience with POCUS, and most did not feel it is important for their fellows’ education. However, there is indeed desire from a few subspecialists at our institution to create a formal curriculum for their fellows. 

On the other hand, about half of subspecialty trainees reported that POCUS is an important part of their future education with fellows desiring a broader array of modalities to learn compared to residents. While currently the majority of POCUS is being performed by fellows in EM, CCM, and cardiology, fellows from a number of other specialties expressed a desire to learn at least one modality of POCUS, typically one specifically related to their field, for example a nephrology fellow desiring to learn renal/bladder US, and an endocrine fellow desiring to learn thyroid US. There appeared to be a considerable disconnect in the perceived future importance and current desire to learn POCUS between subspecialty PDs/APDs and their fellows. It is notable that there was significant overlap in the specialties reported for each cohort. 

Among residents, there was little current practice, and it is unclear whether the current use is taking place under the guidance of trained practitioners such as in the Emergency Department or Intensive Care Unit. While approximately half of residents that responded indicated that POCUS is important to their future practice, almost all of them expressed desire to learn POCUS, with the most desired modalities being abdomen, soft tissue, and eFAST.

The data from out institution indicate that POCUS training for general pediatric and pediatric subspecialty trainees is believed to the important to future practice, and residency/fellowship programs should strongly consider integrating it into their training. First, aspects of clinical ultrasound are being incorporated into most subspecialty content specifications and require competency in POCUS in others. Second, if adult medical practice is a roadmap for the eventual direction of pediatric care, it is likely that POCUS will be further incorporated into the standard evaluation of pediatric patients. By having pediatric practitioners proactively adopt POCUS training, our pediatric patients would benefit from the same standard of care that adult patients receive. Third, with regards to future practice, CPT codes have already been established for reimbursement, making POCUS not only useful for patient care, but financially viable 28. Lastly, a number of trainees reported already using POCUS without formal training, which makes institutions and providers vulnerable to potential medicolegal litigation should these scans be interpreted incorrectly and applied to patient care. 

A major limitation of this study is the response rate to the survey, specifically from the trainees, totaling about 1/3 of possible respondents. Another limitation is that this was performed at a single institution and may not reflect attitudes nationally across other pediatric centers. Also, results of the study may be skewed by those willing to respond. For example, those who currently have ultrasound curricula and those who desire to learn POCUS may have been more likely to respond to the survey, as evidenced by the fact that PEM and PCCM were the two groups with the highest representation in the fellows' cohort. Finally, perceived ambiguity in the survey and inconsistency in respondents’ answers offer some confounding, such as when one cardiology fellow reported that they had a POCUS curriculum, but the other did not.

## Conclusions

While there is no distinct consensus between trainees and their program leaders regarding the importance of POCUS in pediatric general and subspecialty training, there is significant desire at our institution for education and incorporation of POCUS into training and clinical care. As POCUS is a relatively new field in pediatrics, it is not entirely surprising that outside of the specialties that have more traditionally used POCUS, namely EM, CCM, and cardiology, there is not significant use of POCUS in clinical decision-making. Additionally, it is not surprising that trainees who are earlier in their career are more likely than their faculty to desire formal training in POCUS be incorporated into their curricula. There is a clear limitation to this study in that the response rate was less than fifty percent, however the study still demonstrates substantial interest at our institution in learning and incorporating a new clinical skill in an area in which current clinical expertise amongst residents, fellows, and subspecialists is clearly lacking. Pediatric training programs should strongly consider integrating POCUS education into their current curricula for residents/fellows given the current trends in training requirements, future viability of POCUS beyond patient care, and possible medicolegal liability of scans being performed by untrained practitioners. While POCUS applications different among the various subspecialties, this study demonstrates that there is a need to incorporate POCUS education into training. Further studies are needed, including a survey of multiple institutions, to evaluate the generalizability of these findings. Other future studies/projects should focus on the creation of a POCUS curriculum with skills that are generalizable across subspecialties, as well as specialty-specific applications. 

## Conflict of Interest 

The authors have no conflicts of interest to disclose.
